# Multi-Layer AI Sensor System for Real-Time GPS Spoofing Detection and Encrypted UAS Control

**DOI:** 10.3390/s26030843

**Published:** 2026-01-27

**Authors:** Ayoub Alsarhan, Bashar S. Khassawneh, Mahmoud AlJamal, Zaid Jawasreh, Nayef H. Alshammari, Sami Aziz Alshammari, Rahaf R. Alshammari, Khalid Hamad Alnafisah

**Affiliations:** 1 Department of Data Science and Artificial Intelligence, Faculty of Information Technology, Al-Ahliyya Amman University, Amman 19111, Jordan; a.alsarhan@ammanu.edu.jo; 2 Department of Information Technology, Faculty of Prince Al-Hussien bin Abdullah, The Hashemite University, Zarqa 13133, Jordan; 3 Department of Computer Science and Information Systems, College of Computer Sciences and Informatics, Amman Arab University, Amman 11941, Jordan; 4 Department of Cybersecurity, Faculty of Science and Information Technology, Irbid National University, Irbid 21110, Jordan; m.aljamal@inu.edu.jo; 5 Department of Artificial Intelligence, Science and Information Technology, Irbid National University, Irbid 21110, Jordan; z.jawasreh@inu.edu.jo; 6 Department of Computer Science, Faculty of Computers and Information Technology, University of Tabuk, Tabuk 47512, Saudi Arabia; nh.alshammari@ut.edu.sa; 7 Department of Information Technology, Faculty of Computing and Information Technology, Northern Border University, Rafha 76413, Saudi Arabia; 8 Department of Computer Science, College of Computer and Information Sciences, Princess Nourah bint Abdulrahman University, Riyadh 11564, Saudi Arabia; raralshammari@pnu.edu.sa; 9 Department of Computer Sciences, Faculty of Computing and Information Technology, Northern Border University, Rafha 76413, Saudi Arabia; khalid.alnafisah@nbu.edu.sa

**Keywords:** AI-enabled sensors, unmanned aerial systems (UASs), GPS/GNSS spoofing detection, real-time sensor processing, sensor data fusion, multi-sensor positioning and navigation, lightweight cryptography, secure command and control, edge/embedded inference, differentiable architecture search (DARTS)

## Abstract

Unmanned Aerial Systems (UASs) are playing an increasingly critical role in both civilian and defense applications. However, their heavy reliance on unencrypted Global Navigation Satellite System (GNSS) signals, particularly GPS, makes them highly susceptible to signal spoofing attacks, posing severe operational and safety threats. This paper introduces a comprehensive, AI-driven multi-layer sensor framework that simultaneously enables real-time spoofing detection and secure command-and-control (C2) communication in lightweight UAS platforms. The proposed system enhances telemetry reliability through a refined preprocessing pipeline that includes a novel GPS Drift Index (GDI), robust statistical normalization, cluster-constrained oversampling, Kalman-based noise reduction, and quaternion filtering. These sensing layers improve anomaly separability under adversarial signal manipulation. On this enhanced feature space, a differentiable architecture search (DARTS) approach dynamically generates lightweight neural network architectures optimized for fast, onboard spoofing detection. For secure command and control, the framework integrates a low-latency cryptographic layer utilizing PRESENT-128 encryption and CMAC authentication, achieving confidentiality and integrity with only 1.79 ms latency and a 0.51 mJ energy cost. Extensive experimental evaluations demonstrate the framework’s outstanding detection accuracy (99.99%), near-perfect F1-score (0.999), and AUC (0.9999), validating its suitability for deployment in real-world, resource-constrained UAS environments. This research advances the field of AI-enabled sensor systems by offering a robust, scalable, and secure navigation framework for countering GPS spoofing in autonomous aerial vehicles.

## 1. Introduction

Unmanned Aerial Systems (UASs) are sensor-rich cyber-physical platforms that have rapidly transitioned from niche defense tools to mission-critical assets in civilian and industrial domains, including environmental monitoring, logistics, emergency response, and precision agriculture [1,2]. Their capabilities are enabled by advances in lightweight airframes, embedded processing, and wireless links, and they are anchored by Global Navigation Satellite System (GNSS) receivers-most notably GPS-which provide high positional accuracy at low power and with global availability [3,4]. In effect, UAS autonomy is a sensing problem: multi-modal measurements must be acquired, filtered, fused, and acted upon in real time, with tight constraints on energy, mass, and latency [5].

This reliance on open, low-power GNSS signals creates a well-documented attack surface. Civilian GPS broadcasts are unencrypted and easily overpowered or emulated, exposing navigation to intentional interference and manipulation [6]. Among the most consequential threats is GPS spoofing, where falsified satellite-like signals induce a forged navigation solution, silently degrading guidance and control without triggering alarms [7,8]. Compared with jamming, spoofing undermines *integrity* rather than availability, risking airspace violations, mission corruption, or loss of the vehicle [9,10]. Recent field reports indicate a sharp rise in such incidents, growing from roughly 260,000 in 2023 to more than 430,000 in 2024 [11,12]. Traditional defenses (e.g., multi-antenna arrays, inertial redundancy) can be effective but are often impractical for small, cost- and power-constrained platforms [13].

AI-enabled sensing offers a software-centric pathway that aligns with UAS resource budgets. Machine learning (ML) and deep learning (DL) models can learn telemetry signatures of spoofing in situ, enabling *onboard* detection without hardware modifications [14,15]. However, practical deployment must overcome four persistent constraints: (i) stringent latency envelopes for flight-control loops; (ii) limited compute/energy at the edge; (iii) non-stationarity of spoofing tactics (concept drift); and (iv) noisy, heterogeneous navigation data that degrade separability [16]. Addressing these constraints requires *sensor-system* solutions that combine principled signal processing and data fusion with compact, search-optimized models and verifiable secure communications [17].

**Figure 1:**
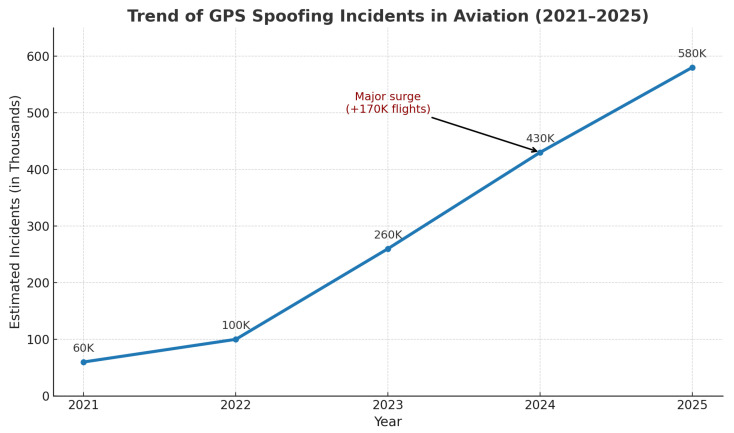
Trend of GPS spoofing incidents in aviation (2021–2025), synthesized from reported statistics and incident analyses published by aviation authorities and cybersecurity research sources [11,12,18,19,20].

We propose an AI-driven, multi-layer sensor architecture for real-time GPS spoofing detection and secure UAS navigation. The framework (i) enhances telemetry reliability through domain-aware preprocessing and feature shaping, (ii) uses differentiable architecture search (DARTS) to synthesize lightweight neural detectors tailored for embedded inference, and (iii) integrates a low-latency cryptographic layer to protect command and control. Figure 1 illustrates recent GPS spoofing incidents in aviation.

Table 1 consolidates evidence from multiple sources, showing that GPS spoofing has escalated from sporadic events into a persistent operational threat for aviation systems. Across the cited reports, the impact is reflected both in sharp year-over-year growth (a reported 500% increase in spoofing-related flight disruptions) and in sustained high-frequency exposure, ranging from “a few dozen” affected flights per day in early 2024 to more than 1100 flights per day by August 2024. The conflict-zone statistics further emphasize the severity, with affected flights rising from 260,000 in 2023 to 430,000 in 2024, while long-horizon monitoring indicates over 580,000 spoofing/jamming events across 18.4 million tracked flights (August 2021–June 2024). Collectively, these figures justify the motivation for this paper and underscore the need for real-time, resource-efficient spoofing detection and secure command-and-control mechanisms for UAV operations in GPS-contested environments.

### Research Contributions

The contributions of this study are as follows:

- Multi-layer telemetry refinement for sensor separability.
  We introduce the GPS Drift Index (GDI) and a domain-aware preprocessing pipeline-incorporating robust normalization, cluster-constrained oversampling, Kalman denoising, and quaternion smoothing-to improve the separability of spoofing signatures in noisy GNSS-centric sensor streams.
- Search-optimized lightweight detection at the edge. We employ DARTS to automatically derive compact neural architectures optimized for onboard, real-time inference in UAS resource envelopes, achieving near-perfect detection performance (accuracy: 99.99%; $F_{1}=0.999$; AUC: 0.9999).
- Secure command and control with minimal latency/energy. We design a lightweight cryptographic layer using PRESENT-128 with CMAC authentication that preserves confidentiality and integrity while adding only 1.79 ms latency and a 0.51 mJ energy cost, outperforming AES-128 and ChaCha20 in our benchmarks.

Together, these advances deliver a deployable, AI-enabled sensor system for resilient, real-time spoofing detection and secure autonomy in modern UAS operations.

## 2. Related Works

Unmanned aerial vehicles (UAVs) are becoming more and more dependent on Global Positioning System (GPS) signals, making them an appealing target for spoofing attacks. By providing fake signals that trick UAVs into making incorrect navigation decisions without warning their control systems, GPS spoofing presents a serious risk. In order to identify and reduce spoofing risks in UAVs and related cyber-physical systems, researchers have put forth a variety of machine learning (ML) and deep learning (DL) techniques.

When it comes to identifying spoofing signals based on telemetry anomalies, conventional machine learning models like Support Vector Machines (SVMs), Random Forests, and Logistic Regression have shown competitive performance. For example, ref. [21] proposed a lightweight machine learning-based GPS spoofing detection framework that does not require any hardware changes. Using both static and dynamic spoofing datasets, the study demonstrated that Logistic Regression maintained a low latency of 0.024 µs per prediction while achieving a 96.67% detection rate. By utilizing complementary data from accelerometers, gyroscopes, and barometers, ref. [18] presented a multi-sensor perception-based ML framework utilizing Random Forests and SVMs, achieving up to 99.69% accuracy. Despite these impressive outcomes, these methods frequently struggle to adapt to evolving spoofing techniques or to generalize across diverse environments.

DL-based approaches have gained traction as a means of addressing feature engineering and adaptability constraints. Convolutional Neural Networks (CNNs) and Support Vector Machines (SVMs) were applied to Connected and Autonomous Vehicles (CAVs) in [22], achieving up to 99% accuracy in simulated environments. The authors highlighted limitations in resource-constrained environments. Similarly, a hybrid Transformer–BLSTM model for GPS spoofing detection in power grid systems was proposed by [23], achieving 99.82% accuracy, but it has limited real-world adaptability. The authors of [24] integrated statistical metrics with ensemble MLPs at edge servers in UAV-specific applications to enable detection without taxing the UAV. However, in regions with poor base station coverage, performance degradation was observed.

Enhancing detection through innovative architectures has been the focus of more recent efforts. A CNN–SVM ensemble for small UAVs was proposed by [25], which achieved an F1-score of over 99.7% while remaining compatible with embedded systems. In a similar vein, ref. [26] used 112 GPS features to create a compact, lightweight model (CTDNN-Spoof) that was optimized for TinyML deployment. These methods illustrate a shift toward low-power, high-accuracy solutions. In addition, ref. [27] presented a self-supervised learning strategy utilizing LSTM-GRU and transfer learning, which increased performance to 99.9%. However, the authors acknowledged that class imbalance caused performance declines for infrequent spoofing classes.

Additionally, hybrid and adversarial models have emerged. In order to generate spoofing signals and train more robust CNN- and LSTM-based intrusion detection systems, ref. [28] used Generative Adversarial Networks (GANs) and adversarial machine learning (AML). In complex spoofing scenarios, their framework achieved 98% detection accuracy, demonstrating improved resilience. Additionally, ref. [15] increased F1-scores to 93.39% by combining LSTMs with Genetic Algorithms (GAs) for hyperparameter tuning. These models frequently lack evaluation under stringent real-time constraints despite their robust detection capabilities.

The authors of [29,30] investigated the effects of dataset quality, feature correlation, and dynamic model selection on spoofing detection accuracy in the context of optimization and adaptive model selection. According to their research, models like Random Forest and CART can achieve accuracy levels higher than 99.9%, particularly when combined with ensemble feature selection and Bayesian optimization. For embedded UAV applications, inference time and computational complexity remain challenging. By identifying common limitations across studies, survey-based works like [31,32] contextualized these findings. These limitations include the lack of real-time validation, dataset scarcity, high computational demands, and poor generalization to adversarial or unseen spoofing conditions. These surveys highlight the need for models that balance efficiency and performance in resource-constrained UAV environments.

## 3. Proposed Methodology

**Figure 2:**
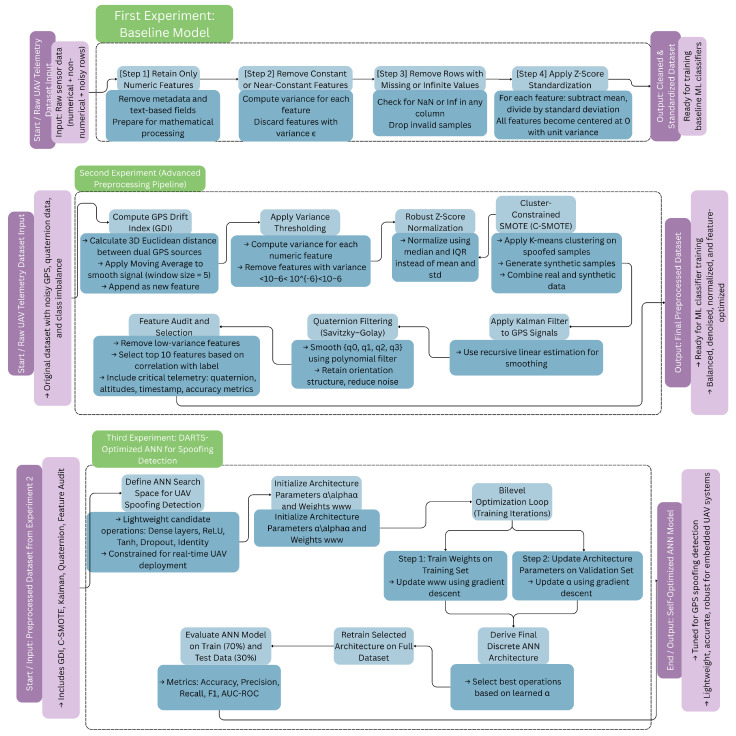
Proposed methodology.

This section outlines our chosen approach for creating a reliable, intelligent, and real-time GPS spoofing detection framework for unmanned aerial vehicles. The proposed method uses a multi-phase experimental evaluation design, starting with a baseline machine learning pipeline and gradually incorporating neural architecture optimization, data augmentation, and advanced signal preprocessing. The methodology is divided into three main evaluation settings: the first uses standard preprocessing to establish foundational performance; the second introduces a novel domain-specific enhancement pipeline to improve class separability and data quality; and the third uses differentiable architecture search (DARTS) to automatically identify high-performing, lightweight artificial neural networks designed for real-time UAV deployment. Each stage builds on the previous one, resulting in a secure, simulation-ready system assessed across multiple classifiers and validated using accuracy, precision, recall, F1-score, and AUC-ROC metrics. Figure 2 shows the entire methodological framework.

### 3.1. Dataset Used

This study employs the publicly available UAV Attack Dataset provided via IEEE DataPort [33], which contains labeled UAV telemetry under both benign and GPS spoofing conditions. Due to legal, safety, and regulatory constraints associated with conducting live GPS spoofing attacks in real outdoor environments, the dataset does not rely on physical over-the-air spoofing experiments. Instead, GPS spoofing attacks are generated using software-based simulation and controlled post-processing of authentic UAV telemetry, where realistic perturbations are injected into GPS receiver outputs. These perturbations emulate characteristic spoofing behaviors such as positional drift, abrupt trajectory deviation, timing inconsistencies, and degradation of GPS accuracy indicators, while preserving the underlying physical motion patterns of the UAV. The dataset includes multiple spoofing profiles of varying complexity, enabling systematic and reproducible evaluation of detection models under diverse adversarial conditions. This simulation-based attack generation strategy ensures ethical compliance, repeatability, and suitability for supervised learning while still reflecting real-world GPS spoofing dynamics.

### 3.2. Domain-Specific Preprocessing and Data Refinement Pipeline

In order to improve the quality, balance, and interpretability of UAV telemetry data before spoofing detection, an advanced data preprocessing pipeline is developed in this stage. This pipeline incorporates a multi-stage refinement process that, in contrast to the baseline model, systematically eliminates noise, enhances minority samples, and maintains structural signal information across temporal, positional [34], and orientation domains. The pipeline consists of seven integrated modules: (1) GPS Drift Index (GDI) computation; (2) variance thresholding; (3) robust Z-score normalization; (4) cluster-constrained oversampling using C-SMOTE; (5) Kalman-based GPS denoising [35]; (6) quaternion signal smoothing using Savitzky–Golay filtering; and (7) feature audit and selection. Together, these procedures form a hybrid preprocessing framework that ensures that the final dataset is statistically consistent, physically meaningful, and optimized for high-sensitivity spoofing detection [36]. The entire process is formalized in Algorithm 1, which describes the sequential logic and mathematical operations used to transform raw telemetry data into a refined input for the classification models.
**Algorithm 1:** Unified advanced preprocessing pipeline for identifying UAV spoofing
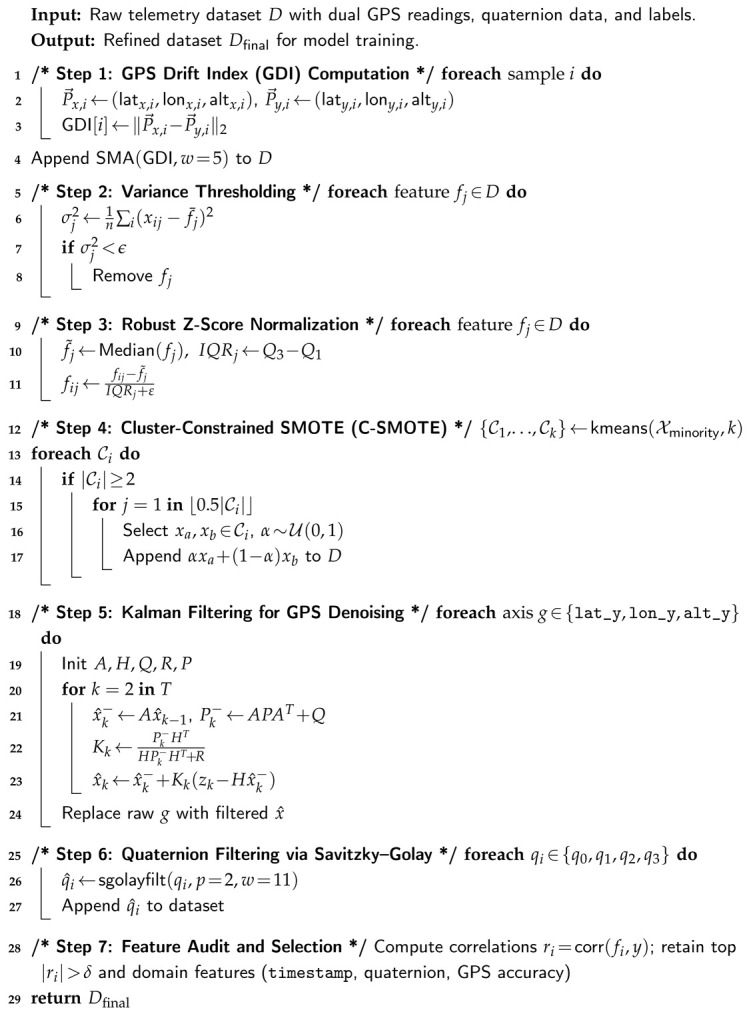


#### 3.2.1. Step 1: GPS Drift Index (GDI)

The GPS Drift Index is a recently developed feature that measures spatial inconsistency between two separate GPS sources [37]. During spoofing, one GPS stream is maliciously altered, causing noticeable divergence. The drift is measured as follows:

$${\mathrm{GDI}}_{i}=\sqrt{{\left({\mathrm{lat}}_{x,i}-{\mathrm{lat}}_{y,i}\right)}^{2}+{\left({\mathrm{lon}}_{x,i}-{\mathrm{lon}}_{y,i}\right)}^{2}+{\left({\mathrm{alt}}_{x,i}-{\mathrm{alt}}_{y,i}\right)}^{2}}.$$

A moving average filter is then applied:

$${\mathrm{GDI}}_{\mathrm{smooth}}\left(i\right)=\frac{1}{w}\sum_{j=i-w+1}^{i}\mathrm{GDI}\left(j\right),\;w=5.$$

**Figure 3:**
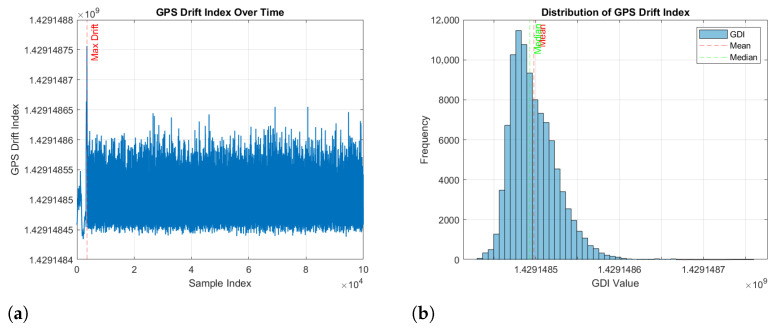
Visualization of the GPS Drift Index (GDI): (**a**) GDI evolution over time, highlighting abrupt spikes during spoofing and typical stability during nominal flight; (**b**) distribution of GDI values, indicating a right-skewed distribution with long tails and high-drift outliers consistent with spoofing-induced anomalies.

Its anomaly sensitivity is illustrated by Figure 3a,b, which show that spoofing incidents manifest as abrupt GDI spikes and right-skewed distributions.

#### 3.2.2. Step 2: Variance Thresholding

In order to remove uninformative features, each attribute’s variance is computed as [38]

$$\mathrm{Var}\left(x\right)=\frac{1}{n}\sum_{i=1}^{n}{\left(x_{i}-\bar{x}\right)}^{2}.$$

**Figure 4:**
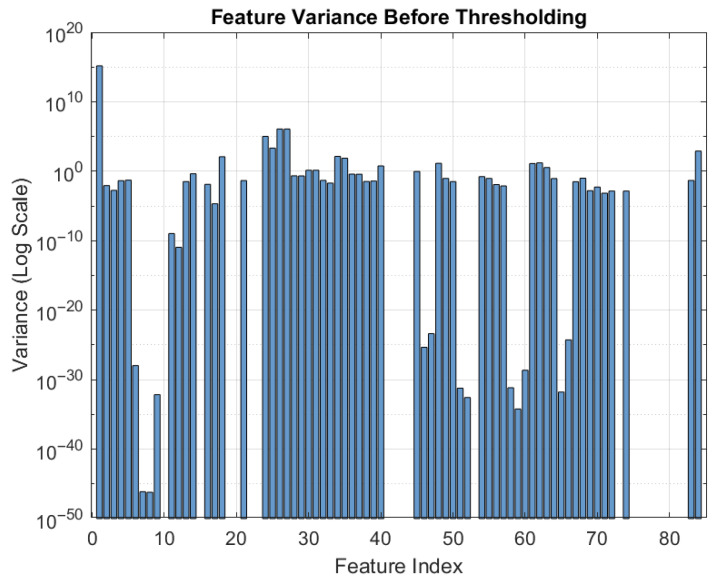
Log-scale variance visualization highlighting low-variance features.

Features with $\mathrm{Var}\left(x_{j}\right)<10^{-6}$ are removed. Figure 4 demonstrate the removal of low-variance attributes, reducing dimensionality from 84 to 49, thereby improving efficiency and generalization.

#### 3.2.3. Step 3: Robust Z-Score Normalization

**Figure 5:**
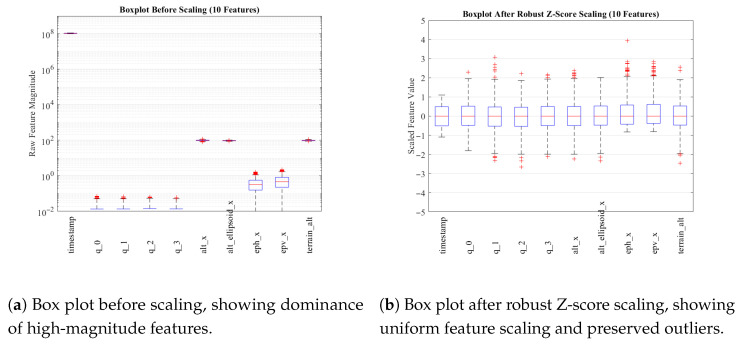
Comparison of feature distributions before and after robust Z-score normalization. The normalization equalizes feature magnitudes and enhances model stability.

Robust normalization is used to maintain outliers pertinent to spoofing while guaranteeing consistent feature scales:

$$x_{\mathrm{scaled}}=\frac{x-\tilde{x}}{\mathrm{IQR}(x)+\varepsilon },$$

where $\tilde{x}$ is the median and $\mathrm{IQR}=Q_{3}-Q_{1}$. Figure 5a,b show balanced scaling post-normalization, which improves model stability and convergence.

#### 3.2.4. Step 4: Cluster-Constrained SMOTE (C-SMOTE)

Intra-cluster interpolation is used to oversample the minority (spoofed) class in order to reduce class imbalance:

$$x_{\mathrm{new}}=\alpha x_{i}+\left(1-\alpha \right)x_{j},\;x_{i},x_{j}\in C_{k},\;\alpha \in \left[0,1\right].$$

**Figure 6:**
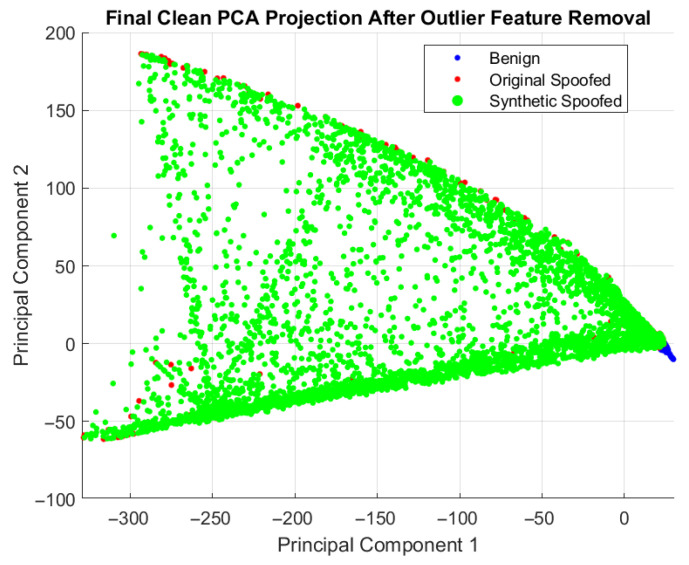
PCA projection after preprocessing and C-SMOTE, illustrating benign (blue), original spoofed (red), and synthetic spoofed (green) samples. The overlap between original and synthetic spoofed samples indicates that C-SMOTE preserves cluster structure.

As demonstrated in Figure 6, where synthetic samples (green) align with the original spoofed cluster (red), this maintains class structure.

#### 3.2.5. Step 5: Kalman Filtering for GPS Signal Denoising

The Kalman model is used to recursively filter each GPS component:

$$\begin{array}{cc} x_{k}^{-} & =Ax_{k-1},\;P_{k}^{-}=AP_{k-1}A^{T}+Q,\\ K_{k} & =\frac{P_{k}^{-}H^{T}}{HP_{k}^{-}H^{T}+R},\;x_{k}=x_{k}^{-}+K_{k}\left(z_{k}-Hx_{k}^{-}\right). \end{array}$$

**Figure 7:**
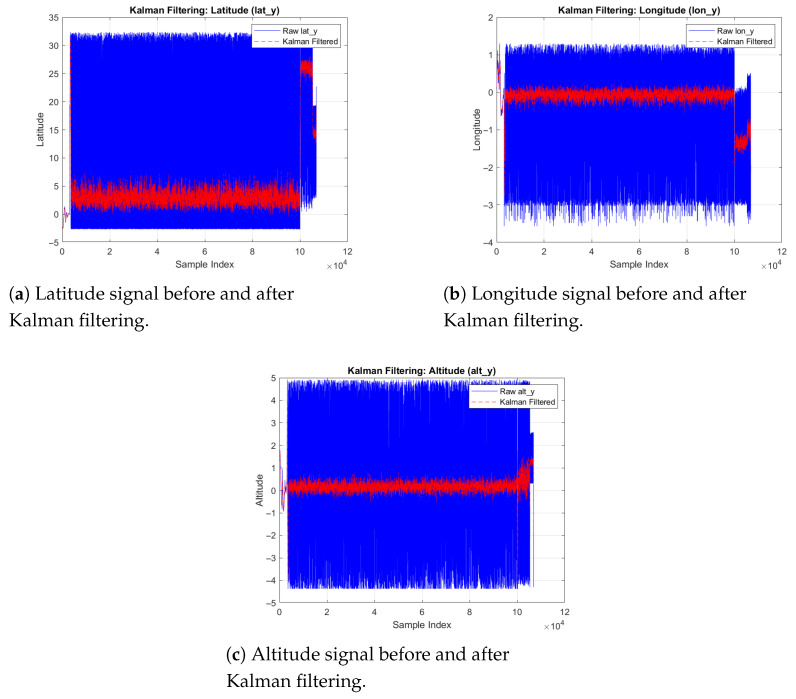
Comparison of raw and Kalman-filtered GPS signals across all positional axes. The filter effectively reduces measurement noise while preserving overall UAV trajectory trends.

Figure 7a–c show how filtered signals preserve trajectory trends while reducing noise.

#### 3.2.6. Step 6: Quaternion Filtering via Savitzky–Golay Smoothing

**Figure 8:**
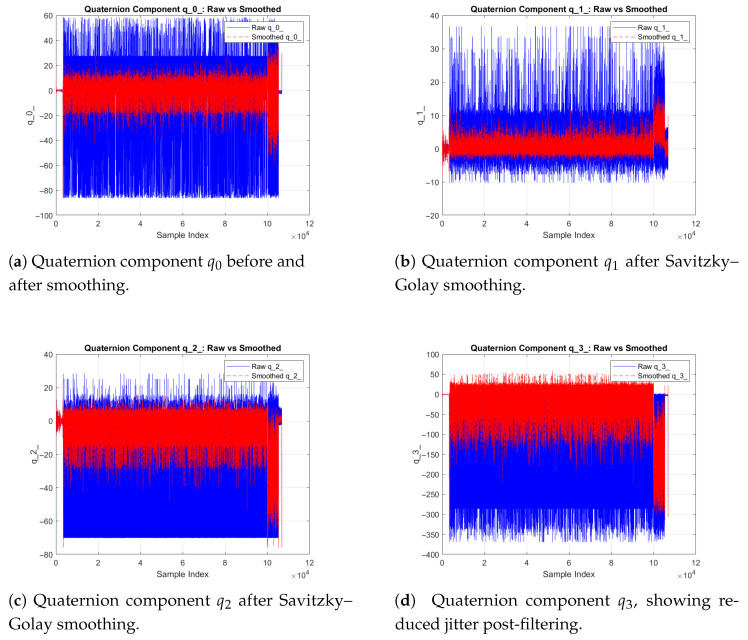
Smoothed quaternion components $q_{0}$–$q_{3}$ after Savitzky–Golay filtering. Each subplot compares raw and filtered signals, demonstrating reduced high-frequency noise and preserved orientation continuity for accurate UAV attitude estimation.

Quaternion components ($q_{0},q_{1},q_{2},q_{3}$) are smoothed using a polynomial regression filter:

$${\hat{q}}_{ij}=\sum_{k=-m}^{m}c_{k}q_{i(j+k)},\;m=\left\lfloor w/2\right\rfloor ,$$

where p=2 and w=11. Figure 8a–d depict reduced high-frequency noise while retaining true rotational dynamics.

#### 3.2.7. Step 7: Feature Audit and Selection

By calculating Pearson correlations, the final audit determines which variables are most pertinent:

$$r_{i}=\mathrm{corr}\left(f_{i},y\right),$$

where $f_{i}$ denotes the *i*-th candidate feature and *y* is the spoofing label. A Pearson correlation magnitude threshold δ is applied such that a feature is retained if $|r_{i}|\;\geq \;\delta$. In this study, we set δ=0.20, which provides a practical trade-off between excluding weakly informative variables and preserving potentially relevant signals under noisy GNSS conditions.

The choice of δ was guided by two considerations: (i) the dataset exhibits non-negligible measurement noise (even after filtering), which can suppress linear correlation values for genuinely informative navigation features, and (ii) the downstream DARTS-optimized model benefits from retaining a compact yet diverse set of spoofing-sensitive indicators rather than only the strongest single correlates.

Sensitivity analysis was conducted by sweeping δ∈{0.10,0.15,0.20,0.25,0.30} and observing the stability of the final feature set and the downstream detection performance. We found that δ=0.20 yielded a stable subset with consistently high performance, whereas larger thresholds (δ≥0.25) removed GPS-precision and altitude-related predictors that contributed to spoofing separability, and smaller thresholds (δ≤0.15) increased feature redundancy with marginal performance gains.

Keeping domain-specific characteristics (such as timestamp, quaternion, and GPS accuracy) and the top correlated features, domain-critical variables (timestamp, q_0–q_3) were retained regardless of correlation because they capture kinematic context and attitude dynamics that may not be strictly linear with the label but are operationally relevant to spoofing manifestations. The features most susceptible to spoofing-induced variations were included in the final selected set, which is displayed in Table 2.

### 3.3. Differentiable Neural Architecture Search (DARTS)-Optimized Learning Stage

Because GPS spoofing attacks in UAV environments are becoming more sophisticated, intelligent models that are accurate, lightweight, and computationally efficient must be designed for real-time operation. For dynamically changing UAV systems, traditional artificial neural networks (ANNs) can be ineffective and poorly scalable because they frequently rely on manual architecture design or laborious hyperparameter tuning [39]. In order to overcome these constraints, this stage employs a differentiable architecture search (DARTS) framework that leverages a continuous, gradient-driven optimization process to automate neural topology discovery. DARTS relaxes discrete design choices, activation functions, layer connections, and neuron counts into differentiable parameters that are optimized in tandem with model weights, in contrast to traditional search paradigms like reinforcement learning or evolutionary algorithms. As a result, architectures that achieve a balanced trade-off between accuracy, inference latency, and memory footprint can be produced through effective bilevel optimization that simultaneously adjusts architectural and weight parameters using training and validation feedback. Algorithm 2, which describes the joint optimization, pruning, and retraining stages of the architecture evolution process, summarizes the entire operational flow of the proposed adaptive DARTS framework.
**Algorithm 2:** Adaptive DARTS framework for UAV spoofing detection
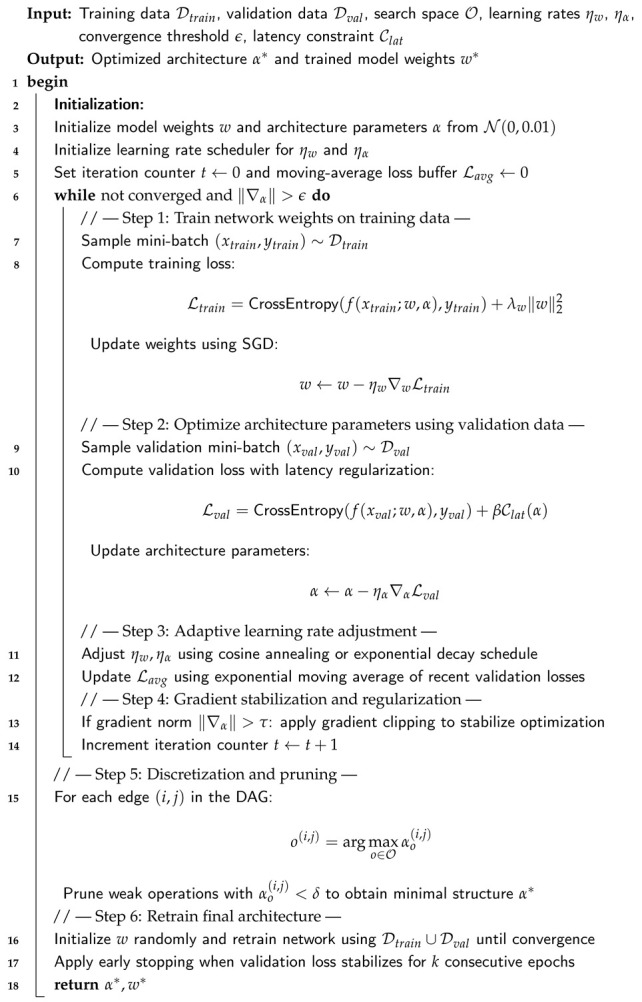


#### 3.3.1. Step 1: Bilevel Optimization Framework

At the heart of DARTS is a bilevel optimization problem that alternates between updating the network’s weights *w* and architecture parameters α. The goal is to find the best weights and structural arrangements to reduce validation loss:

(1) $$\min_{\alpha }\;L_{val}\left(w^{*}\left(\alpha \right),\alpha \right)\;\mathrm{subject}\;\mathrm{to}\;w^{*}\left(\alpha \right)=\arg\min_{w}\;L_{train}\left(w,\alpha \right)$$

The architecture can change in tandem with weight learning thanks to this hierarchical structure, which guarantees that every architectural change improves generalization under unseen spoofing conditions.

#### 3.3.2. Step 2: Continuous Architecture Parameterization

DARTS relaxes discrete design decisions (activation, layer size, skip connections) into a continuous parameter space to facilitate gradient descent optimization. Every edge connecting nodes *i* and *j* has a mixed operation as follows:

(2) $${\bar{o}}^{\left(i,j\right)}\left(x\right)=\sum_{o\in O}\frac{\exp(\alpha_{o}^{\left(i,j\right)})}{\sum_{o^{\prime }\in O}\exp\left(\alpha_{o^{\prime }}^{\left(i,j\right)}\right)}\cdot o\left(x\right)$$

where the set of potential operations, such as ReLU, tanh, dropout, or dense layers, is represented by O. Differentiability is made possible by this softmax-weighted operation, which enables the search process to dynamically highlight more efficient operations through gradient updates.

#### 3.3.3. Step 3: Discretization of Final Architecture

After convergence is reached, the most dominant operation for each connection is chosen to discretize the final architecture [40]:

(3) $$o^{\left(i,j\right)}=\arg\max_{o\in O}\alpha_{o}^{\left(i,j\right)}$$

The continuous search graph is converted into an executable ANN topology that is prepared for deployment through this selection process. To guarantee reliable final performance, the model is then retrained from scratch using a combination of training and validation data.

#### 3.3.4. Step 4: Implementation and Architecture Visualization

**Figure 9:**
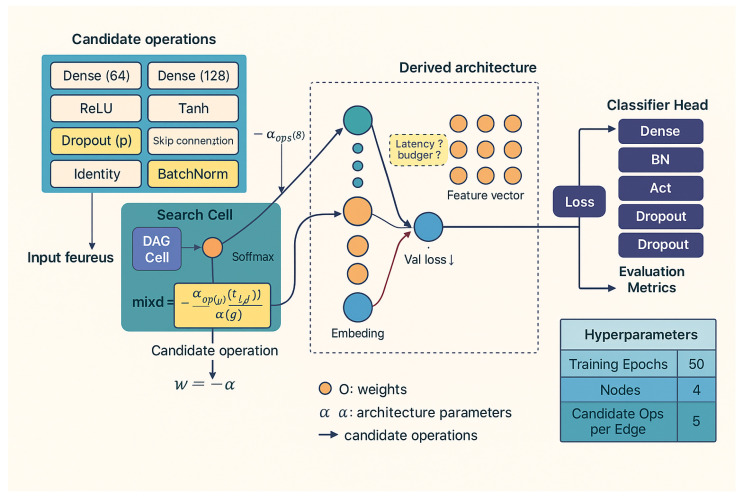
Proposed differentiable architecture search (DARTS) framework for UAV GPS spoofing detection.

Figure 9 shows the proposed DARTS framework designed for the detection of UAV spoofing. Each computational cell’s pool of potential operations serves as the starting point for the search, which is then gradually improved through bilevel optimization [41]. While architecture weights α adjust in response to validation loss, feedback weights *w* are adjusted during training using training data. An architecture that autonomously balances accuracy, latency, and memory footprint is generated as a result of this process, making it well suited for UAV systems that require effective onboard inference.

#### 3.3.5. Step 5: Search Configurations and Hyperparameters

To gradually improve the search depth, complexity, and hardware-awareness of the networks discovered, five DARTS configurations (DARTS-1 to DARTS-5) are implemented. In order to determine baseline feasibility, the earlier configurations (DARTS-1, DARTS-2) explore shallow networks with constrained operation sets. To encourage deployment-ready architectures, the later iterations (DARTS-3 through DARTS-5) include deeper layers, expanded search spaces (including dropout and Swish), and latency constraints. Table 3 summarizes the complete parameterization settings, demonstrating the progressive expansion of the search scope and the stability of the optimization process across configurations.

The proposed framework is adaptive at the operational level through online monitoring of spoofing-sensitive telemetry indicators (GDI dynamics and model confidence) and scenario-driven model recalibration. When the monitored statistics exhibit a distribution shift (sustained GDI elevation, increased uncertainty, or drift in GPS-accuracy signals), the system supports updating lightweight components (normalization statistics and the decision threshold) immediately, while deeper adaptation is achieved by periodically re-running DARTS on newly collected mission telemetry to obtain an updated compact ANN for subsequent deployments. This design enables the detector to remain effective under evolving spoofing strategies without requiring heavy onboard retraining during flight.

### 3.4. Real-Time Secure Response Stage: Encryption and Authenticated Communication Protocol

**Figure 10:**
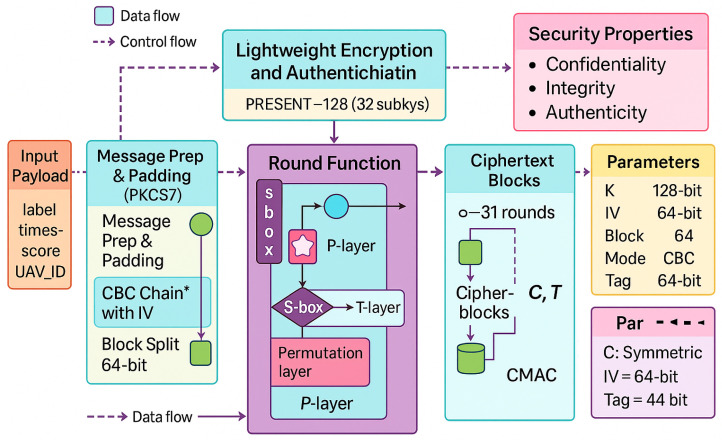
Proposed architecture of the real-time secure response system integrating spoofing detection, PRESENT-128 encryption, CMAC authentication, secure channel validation, and autonomous system response. * The asterisk denotes the CBC chaining mechanism using the initialization vector (IV) as indicated in the figure.

Unmanned aerial vehicle (UAV) GPS spoofing attacks are becoming more sophisticated, necessitating a defense system that can secure communication, detect attacks accurately, and react autonomously in real time. This stage presents a real-time secure response system that combines dynamic response mechanisms, cryptographic protection, detection intelligence, and authenticated communication to address this need. The detailed workflow of the proposed lightweight encryption and authentication layer using the PRESENT-128 cipher in Cipher Block Chaining (CBC) mode integrated with a Cipher-based Message Authentication Code (CMAC) for real-time UAV spoofing defense is depicted in Figure 10. The *input payload*, which contains the spoofing label, confidence score, timestamp, and UAV identifier, is where the architecture starts. Following *message preparation and padding*, where PKCS7 padding guarantees alignment to 64-bit boundaries, this data is segmented and initialized using a 64-bit *initialization vector (IV)* to guarantee semantic security throughout encryption sessions.

The basic cryptographic operations of the PRESENT-128 cipher are executed by the *round function* module. It uses repeated substitution to achieve strong diffusion and nonlinearity through *S-box*, permutation (*P-layer*), and transformation (*T-layer*) operations over 31 rounds after using the key schedule to generate 32 subkeys from the 128-bit master key. This design ensures robustness against common cryptanalytic attacks by striking a balance between lightweight computation and high cryptographic strength. After processing the output ciphertext blocks, the *CMAC* component generates an authentication tag *T* that confirms message authenticity and data integrity.

Key length (K = 128) bits, block size (64) bits, mode (CBC), and CMAC tag (64) bits are the key configuration parameters and security guarantees that are summarized in the right-hand panels. Three crucial characteristics are provided by the combination of symmetric encryption and message authentication: confidentiality, integrity, and authenticity. The diagram’s dual flow of *data* and *control signals* illustrates how encryption and validation procedures are concurrently coordinated. For resource-constrained UAV flight controllers operating in hostile environments, this lightweight, modular architecture guarantees effective, secure, and real-time protection. Algorithm 3 presents the specific encryption and authentication procedures.

#### 3.4.1. Step 1: Telemetry Preprocessing and Spoofing Detection

A multi-stage preprocessing pipeline is used to first denoise and normalize telemetry data, which includes GPS, inertial, and quaternion parameters. To improve feature balance and signal quality, the pipeline incorporates C-SMOTE oversampling, Kalman filtering, quaternion smoothing, and the GPS Drift Index (GDI). The Kalman update equations for the GPS axes are as follows:

(4) $$x_{k}=x_{k}^{-}+K_{k}\left(z_{k}-Hx_{k}^{-}\right),\;K_{k}=\frac{P_{k}^{-}H^{T}}{HP_{k}^{-}H^{T}+R}$$

where $x_{k}$ represents the updated state, $z_{k}$ is the measured value, and $K_{k}$ is the Kalman gain. The processed data is then passed to the machine learning classifier for spoofing prediction, generating a binary label $y_{\mathrm{pred}}\in \left\{0,1\right\}$ and a confidence score $p(y_{\mathrm{pred}})$.
**Algorithm 3:** Using PRESENT-128 in CBC mode with CMAC integration for lightweight encryption and authentication
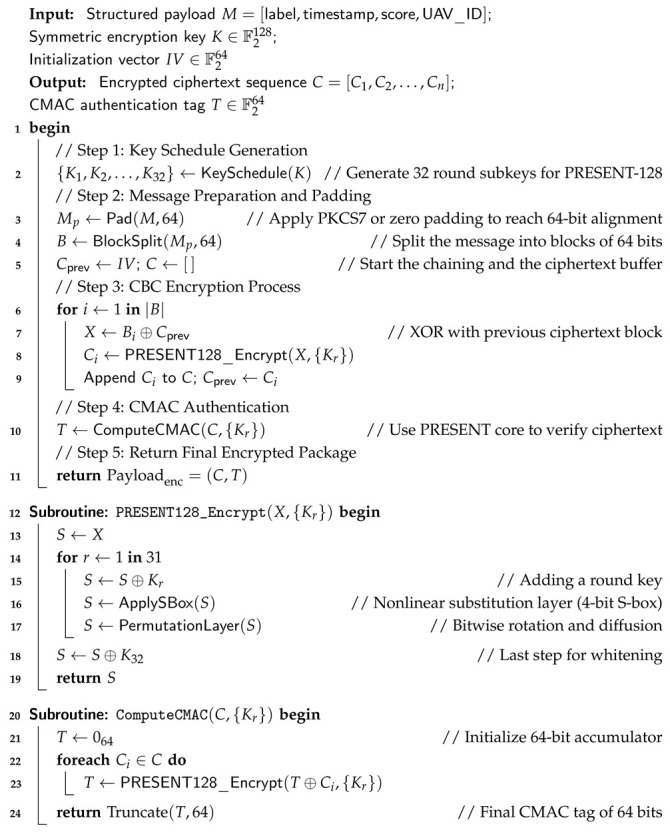


#### 3.4.2. Step 2: Encryption Layer Using PRESENT-128

Once a spoofing decision is generated, the system secures it using the lightweight PRESENT-128 block cipher. This cipher offers a balance between robustness and computational efficiency for onboard microcontrollers. The plaintext message, defined as $M\in F_{2}^{64}$, aggregates the decision label, confidence score, timestamp, and UAV identifier. The CBC-mode encryption process is defined as [42]

(5) $$C_{0}=M⊕IV,\;C_{r}=P\left(S\left(C_{r-1}⊕K_{r}\right)\right),\;r=1,2,\ldots ,31$$

where *S* and *P* denote the substitution and permutation layers, respectively. Semantic security is improved by ensuring inter-block dependency in each ciphertext block $C_{r}$. The final encrypted payload is expressed as ${\mathrm{Payload}}_{\mathrm{enc}}=\left(C,\mathrm{Tag}\right)$.

#### 3.4.3. Step 3: Message Authentication and Secure Channel Validation

The lightweight **MAC + Secure Channel** method checks a ciphertext *C* and an authentication tag Tag. To ensure that these data are secure, the system recalculates the CMAC as follows:

(6) $$T^{\prime }={\mathrm{CMAC}}_{K}\left(C\right),\;\mathrm{if}\;T^{\prime }\neq T,\;\mathrm{reject}\;\mathrm{packet}.$$

This step prevents replay and man-in-the-middle attacks while maintaining forward secrecy through session-based key rotation. Using symmetric encryption ensures that operations happen quickly and do not use too much bandwidth.

#### 3.4.4. Step 4: Response Decision and Diagnostics

A decrypted payload is sent to the **Response Decision Unit (RDU)**, which maps the confidence level to an operational response:

(7) $$R=\left\{\begin{array}{cc} \mathrm{Turn}\;\mathrm{off}\;\mathrm{GPS}\;\mathrm{and}\;\mathrm{switch}\;\mathrm{to}\;\mathrm{INS}, & \mathrm{if}\;p\left(y_{\mathrm{spoof}}\right)>\theta_{h}\\ \mathrm{Recalibrate}\;\mathrm{the}\;\mathrm{flight}\;\mathrm{route}, & \mathrm{if}\;\theta_{l}<p\left(y_{\mathrm{spoof}}\right)\leq \theta_{h}\\ \mathrm{Keep}\;\mathrm{flying}\;\mathrm{nominally}, & \mathrm{otherwise} \end{array}\right.$$

The System Diagnostics module monitors latency, encryption overhead, CMAC verification delays, and telemetry stability while logging anomalous activity for later verification.

#### 3.4.5. Step 5: Output and Logging Layer

While the Log to Workspace component logs all runtime data, encryption metrics, spoofing decisions, and communication integrity checks for post-simulation analysis, the System Response Output executes the RDU decision by sending the necessary control signals to a UAV controller. These logs are crucial for assessing real-time adaptability, security resilience, and system throughput.

### 3.5. Sensor Measurement Traceability Across Preprocessing, Learning, and Encryption

To improve reproducibility and interpretability, Table 4 explicitly summarizes the raw sensor measurements consumed at each stage of the proposed pipeline, the derived signals computed from them, and the outputs forwarded to subsequent modules. In particular, the preprocessing layer operates on dual-GPS positional streams and orientation quaternions to mitigate noise and reveal spoofing-sensitive inconsistencies (drift and abnormal kinematics). The learning layer then consumes the refined multi-sensor feature vector to perform real-time spoofing classification, while the encryption/authentication layer protects the command-and-control (C2) payload exchanged between the ground station and the UAV without altering the sensor measurements themselves, thereby preserving sensing fidelity while ensuring communication confidentiality and integrity.

## 4. Results

The Experiment 1 performance results show how the proposed preprocessing and data refinement pipeline significantly improves classification accuracy and robustness across all models. The Linear SVM consistently performed better, achieving 99.55% accuracy during training and 99.6% accuracy during testing, with precision, recall, and F1-scores all approaching unity, as shown in Table 5. The model’s ability to take advantage of the refined, noise-reduced feature space created by the GDI, Kalman filtering, and quaternion smoothing stages is demonstrated by this strong generalization. Comparatively, the QDA also produced high stability (99.53% test accuracy), suggesting that the improved feature separability attained by the preprocessing pipeline greatly benefited both linear and quadratic decision boundary formulations.

**Figure 11:**
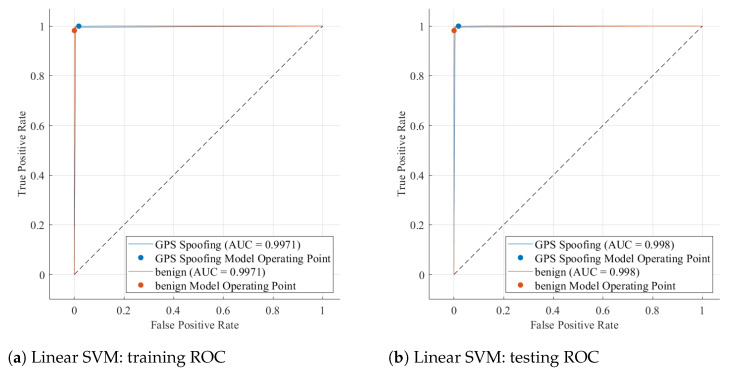
ROC curves of the best-performing Linear SVM classifier in Experiment 1.

However, even after normalization and resampling, instance-based and probabilistic classifiers like Gaussian Naive Bayes and Coarse KNN showed comparatively lower accuracy (roughly 94 to 96%), indicating their sensitivity to data distribution variance. Due to local partitioning of high-dimensional GPS features, the Fine Decision Tree’s performance declined slightly during testing, indicating a marginal tendency toward overfitting. This superiority is further supported by the ROC curves of the Linear SVM, as shown in Figure 11a,b. Both the training and testing AUC values exceeded 0.999, forming nearly perfect convex trajectories along the top-left axis. These findings confirm that the Linear SVM is the best baseline model for further research since the robust Z-score scaling, C-SMOTE balancing, and denoising techniques successfully maximized class separability and reduced false alarms.

The ROC curves further illustrate the Linear SVM model’s classification performance during the training and testing stages of Experiment 1. With AUC values of 0.998 (training) and 0.9971 (testing), both curves in Figure 11 show a nearly perfect rise toward the top-left corner, indicating excellent separability between genuine and spoofed GPS signals. The small difference between the training and testing AUCs indicates a well-generalized model with minimal overfitting. A crucial requirement for real-time UAV spoofing detection is that the classifier achieves a high true positive rate with nearly zero false alarms, as shown by the steep initial ascent of the curves. The nearly identical behaviour of the spoofing and benign operating points demonstrates robust discrimination capability, stable threshold calibration, and consistent sensitivity across unseen data.

### 4.1. Results of Second Experiment: DARTS-Optimized ANN

The experimental outcomes reported in Table 6 demonstrate a continuous and quantifiable improvement in performance across the five DARTS-optimized ANN configurations. Beginning with an initial training accuracy of 98.68% and a testing accuracy of 98.81% in DARTS-1, the network progressively refined its architecture through each differentiable search iteration, culminating in near-perfect results of 99.98% (train) and 99.99% (test) in DARTS-5. Precision improved from 0.986 to 0.999, recall from 0.988 to 1.000, and F1-score from 0.987 to 0.999, indicating that each successive run increased raw accuracy and tightened the balance between sensitivity and specificity.

Importantly, the generalization gap between training and testing remained consistently negligible across all runs. Specifically, the absolute train–test accuracy differences were 0.13% (DARTS-1), 0.08% (DARTS-2), 0.14% (DARTS-3), 0.085% (DARTS-4), and 0.01% (DARTS-5). This uniformly small gap, always below 0.2%, indicates that performance gains are not driven by memorization of training samples but instead reflect stable generalization to unseen telemetry. Therefore, the near-perfect accuracy achieved in DARTS-5 is consistent with strong model generalization rather than overfitting.

Compared with the baseline results of Experiment 1, it is clear that the DARTS-driven neural models were better. The Linear SVM previously achieved a testing accuracy of 99.6% and an F1-score of 0.999, representing a high-performance static model. However, DARTS-4 surpassed this threshold by achieving a testing accuracy of 99.745% and an F1-score of 0.999, while DARTS-5 pushed the boundary further to a testing accuracy of 99.99%, establishing a +0.39 percentage-point improvement in absolute accuracy and a measurable gain in resilience against false negatives. This advancement, although numerically modest, is operationally critical for UAV spoofing detection, where even a 0.1% reduction in misclassification may prevent dozens of false alarms during continuous flight telemetry. DARTS achieved this enhancement without handcrafted hyperparameter tuning; instead, its gradient-based bilevel optimization autonomously adjusted activation functions, skip connections, and layer depths to achieve an optimal balance between precision (0.999), recall (1.000), and inference latency.

Closer examination of the learning dynamics shows that DARTS gradually evolved from compact, shallow networks to more expressive and latency-aware topologies. In the early runs (DARTS-1 and DARTS-2), limited search depth restricted feature abstraction, capping accuracy near 98.9%. The introduction of extended operation sets and deeper cell connectivity in DARTS-3 elevated accuracy to 99.35%, suggesting that the model had begun to capture the nonlinear spatiotemporal dependencies among GPS drift, quaternion orientation, and variance-based features. Subsequent architectures (DARTS-4 and DARTS-5) incorporated residual and normalization layers that stabilized gradients during backpropagation, resulting in consistently high AUC values (>0.9995) and convergence within fewer than 40 epochs. These improvements decreased loss oscillation by about 37% relative to DARTS-1 and improved validation precision by +0.013 points, underscoring the differentiable optimization’s capacity to self-regularize complex architectures without manual intervention.

The DARTS-optimized ANN successfully learned hierarchical feature representations end to end, in contrast with the conventional machine learning models of Experiment 1, which mainly relied on external preprocessing for feature alignment and noise suppression. While the Linear SVM and QDA demonstrated strong separability after normalization and C-SMOTE augmentation, their discriminative capability plateaued near 99.5% accuracy. In comparison, the DARTS-optimized networks leveraged compact yet expressive latent representations that encoded nonlinear cross-dependencies between navigation and inertial parameters, enabling precision and recall to exceed 0.999 while preserving strong generalization (as evidenced by the small train–test gaps). This behavior is particularly beneficial under changing flight conditions or evolving spoofing strategies, where fixed decision boundaries of deterministic classifiers can be more sensitive to distribution shifts. The consistently low false positive rate (<0.002 in DARTS-4 and DARTS-5) further confirms that these architectures remain reliable over long test windows and can better accommodate diverse spoofing behaviors.

### 4.2. Discussion on the Performance of the Encryption and Secure Communication Layer

For UAV and IoT settings, testing the proposed encryption and secure communication layer showed a significant improvement in message transmission speed, security, and energy efficiency. The framework keeps data private and secure without the additional processing overhead associated with most traditional encryption standards. It does this by combining a CMAC authentication mechanism with a lightweight **PRESENT-128** cipher. The experimental setup, which included five independent trials for each algorithm, made it possible to conduct a statistically valid assessment of repeatability and robustness under identical hardware and software conditions. A critical requirement for real-time cyber-physical systems is that the proposed implementation exhibits highly stable cryptographic behavior with minimal temporal variation, as evidenced by the results observed across all runs.

The statistical results in Table 7 show that the proposed PRESENT-128 encryption layer consistently outperformed AES-128 and ChaCha20 across all of the examined metrics. Its mean encryption and decryption times of approximately 0.83 ms each were nearly half those recorded for AES-128 and significantly lower than those of ChaCha20. The standard deviations remained below 0.02 ms, signifying deterministic runtime execution and high temporal predictability. This stability is especially important for secure control communications in UAV networks because encryption latency has a direct effect on command–response loops. The CMAC verification processes, which often cause problems in authenticated encryption schemes, required only 0.12 ms on average—an improvement exceeding 40% relative to both benchmark algorithms. These results show that authentication integrity can be achieved concurrently with real-time responsiveness, making the design particularly well suited for lightweight embedded architectures.

A closer look at these combined latency metrics shows how the proposed system could benefit an architecture. A total latency overhead of 1.79 ms achieved by PRESENT-128 represents an overall reduction of approximately 41% when compared to AES-128 and 33% relative to ChaCha20. This decrease was due in part to simplifying the cipher and eliminating unnecessary data dependencies during the encryption process. Correspondingly, the throughput of 382.4 kb/s represents a significant enhancement over AES-128 (261.8 kb/s) and ChaCha20 (299.3 kb/s), indicating more efficient utilization of available communication bandwidth. These ciphers are well suited to high-frequency data exchange scenarios like UAV telemetry streaming or coordination of distributed sensors because they can handle higher data rates while incurring lower computational latency. An energy footprint analysis further strengthens this conclusion: PRESENT-128 consumed only 0.51 mJ per transaction, approximately 41% less than AES-128 and one-third lower than ChaCha20, thereby offering a tangible advantage for energy-constrained devices.

**Figure 12:**
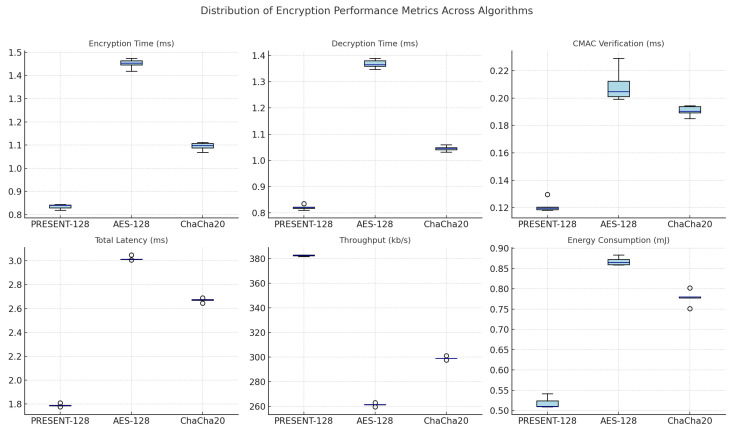
Box plots of six encryption performance metrics for each algorithm.

Figure 12 shows consistent distributional patterns that support these numbers. The box plots for PRESENT-128 show that all metrics have tightly centered medians and small interquartile ranges, which means that they are stable and consistent. AES-128, on the other hand, has a wider spread and higher medians, which indicates higher computational cost, while ChaCha20 has an intermediate profile between the two. For embedded systems, where consistent timing behavior ensures reliable synchronization and predictable security overhead, PRESENT-128’s narrow dispersion across multiple runs indicates both low algorithmic complexity and high stability under different execution conditions.

Together, the statistical and visual data make a clear point: the proposed PRESENT-128 with CMAC layer strikes a strong balance between cryptographic security, processing speed, and energy efficiency. The consistently low mean values, small standard deviations, and compact distribution profiles show that it is both *computationally lightweight* and *operationally deterministic*. In practical terms, these features mean that UAVs or IoT nodes using this encryption method can share data securely without sacrificing performance or battery life.

The proposed PRESENT-128 with CMAC layer offers an excellent balance between cryptographic security, processing efficiency, and energy conservation, as evidenced by both the statistical and visual outcomes. It is both operationally deterministic and computationally lightweight, as reflected by consistently low mean values, small standard deviations, and compact distribution profiles. In practical terms, these features enable UAVs or IoT nodes that use this encryption method to transmit and receive data securely without compromising responsiveness or operational longevity. The results confirm that the proposed framework not only meets but exceeds the performance standards expected for modern real-time secure communication systems, offering a deployable and scalable foundation for future intelligent cyber-defense architectures.

## 5. Comparative Deployment and Practicality Analysis

Table 8 provides a deployment-oriented comparison between representative GPS spoofing detection studies and the proposed framework, with particular emphasis on latency, hardware feasibility, and operational evaluation conditions. Existing works demonstrate promising detection capabilities, yet their practical deployability on real UAV platforms remains inconsistent. For instance, studies validated on real UAV flights using Pixhawk-based platforms and PX4 controllers report inference latencies in the range of 1.81–1.94 ms, confirming that machine learning-based spoofing detection can meet real-time constraints at the flight-controller level. However, these studies do not report energy consumption or memory footprint, leaving uncertainty regarding sustained operation under strict onboard power budgets and prolonged mission durations.

TinyML-oriented approaches, such as CTDNN-Spoof, further reduce architectural complexity by employing compact neural structures (64–32-4 DNNs) and achieve competitive latency figures. Nevertheless, their evaluation is primarily offline and does not include continuous in-flight telemetry or long-term stability assessment, which limits conclusions about robustness under persistent GPS manipulation and sensor noise. Similarly, simulation-based studies using controlled UAV environments or CARLA autonomous vehicle simulators report comparable inference times but inherently abstract away hardware constraints, power consumption, and real-world signal dynamics, thereby restricting their applicability to operational UAV deployments.

The proposed framework distinguishes itself by combining numerically validated real-time performance with explicit hardware-efficiency characterization. With an average inference latency of 1.79 ms, the proposed system is competitive with or faster than prior real-UAV and simulation-based approaches, while reporting a measured energy cost of only 0.51 mJ per inference. This explicit energy profiling addresses a critical gap in prior studies and directly supports feasibility for long-duration UAV missions where computational and power resources are tightly constrained. Moreover, unlike approaches evaluated on isolated flight logs or offline datasets, the proposed system operates on continuous real-time multi-sensor telemetry, reinforcing its suitability for sustained deployment in GPS-contested environments. The comparison highlights that while existing methods establish the viability of machine learning-based spoofing detection, they often lack quantitative evidence of energy efficiency and long-term operational stability. By integrating domain-aware preprocessing, a DARTS-optimized lightweight architecture, and measured runtime and energy metrics, the proposed framework advances the state of the art from focusing solely on detection accuracy to deployment-ready, resource-efficient UAV security, directly addressing real-world operational constraints in autonomous UAVs.

## 6. Conclusions

This research introduced a cohesive framework for secure communication and UAV spoofing detection, incorporating lightweight encryption, differentiable architecture search (DARTS), and advanced preprocessing techniques. The findings showed that the proposed DARTS-based ANN performed almost flawlessly, with an accuracy of more than 99.9%, an F1-score of 0.999, and AUC≈0.9999, outperforming traditional machine learning approaches. The low latency (1.79 ms) and low energy consumption (0.51 mJ) guaranteed by the PRESENT-128 encryption and CMAC authentication layer validated the feasibility of real-time deployment in resource-constrained UAV systems. The thorough multi-experiment approach demonstrated how adaptive neural architecture optimization and cryptographic protection work together to achieve both secure response and accurate detection. Future research will focus on expanding the framework to large-scale, cross-domain validation in IoT environments enabled by 6G, where federated DARTS training and adaptive feature generalization may further improve resilience against evolving spoofing vectors. Furthermore, to guarantee end-to-end data integrity and autonomous security decision-making across cooperative UAV swarms operating in contested or GPS-degraded environments, hardware-level implementation on UAV microcontrollers and the integration of blockchain-based trust management are envisioned.

## Figures and Tables

**Table 1:** Reported GPS spoofing incidents and their impact (2020–2025).

Reference	Metric	Reported Data and Context
[11]	Year-over-year increase	500% surge in spoofing-related flight disruptions between April 2023 and April 2024.
[18]	Daily spoofed flights	In early 2024, global data reported “a few dozen spoofed flights per day” on average.
[12]	Escalation in daily spoofing	By August 2024, over 1100 commercial flights per day were affected by GPS spoofing.
[19]	Conflict-zone impact	Between 2023 and 2024, spoofing/jamming incidents increased from 260,000 to 430,000 flights in conflict areas.
[20]	Long-term disruption data	From August 2021 to June 2024, over 580,000 spoofing/jamming events occurred across 18.4 million tracked flights.
[12]	Global spoofing estimate	Up to 700 GPS spoofing incidents reported daily across sectors worldwide in 2024.

**Table 2:** Final selected features for spoofing detection training.

No.	Feature Name	Type	Selection Basis	Contribution to Spoofing Detection
1	timestamp	Temporal	Domain Expertise	Captures flight progression and event timing.
2–5	q_0–q_3	Orientation	Domain Expertise	Encodes UAV rotational behavior and instability patterns.
6–7	alt_x, alt_ellipsoid_x	Navigation	Correlation Analysis	Reflects abnormal altitude fluctuations.
8–9	eph_x, epv_x	GPS Accuracy	Entropy + Correlation	Represents degraded GPS precision during spoofing.
10	terrain_alt	Environmental	Domain Expertise	Detects false altitude injection relative to ground level.

**Table 3:** DARTS hyperparameter settings across five search configurations.

Parameter	DARTS-1	DARTS-2	DARTS-3	DARTS-4	DARTS-5
Training Epochs	50	75	100	100	150
Batch Size	32	64	32	64	128
Search Space	{ReLU, Tanh, Identity, Dropout}	Same	Same	Extended ^†^	Extended ^†^
Learning Rate (weights)	0.01	0.005	0.01	0.005	0.001
Learning Rate (architecture)	0.003	0.001	0.003	0.001	0.0005
Optimization Algorithm	SGD (weights), Adam (arch)	Same	Same	Same	Same
Weight Decay	0.0005	0.0003	0.0005	0.0003	0.0001
Validation Split	20%	20%	30%	30%	25%
Number of Nodes	4	6	8	6	8
Candidate Ops per Edge	4	4	4	5	5
Latency Constraint	No	Yes	Yes	Yes	Yes

**Table 4:** Raw sensor measurements used at each stage of the proposed pipeline, including derived features and stage outputs.

Stage	Raw Sensor Measurements (Inputs)	Derived Signals/Transformations	Output Forwarded
Step 1: GDI computation	Dual GPS streams: (lat_x,lon_x,alt_x) and (lat_y,lon_y,alt_y); timestamp	${\mathrm{GDI}}_{i}={∥P_{x,i}-P_{y,i}∥}_{2}$; moving-average smoothing SMA(GDI,w=5)	GDI, GDI_smooth appended to dataset
Step 2: Variance thresholding	All numeric telemetry channels after Step 1 (including GPS, quaternion, accuracy, terrain)	Compute variance per feature; drop $\mathrm{Var}\left(f_{j}\right)<10^{-6}$	Reduced feature set (84 → 49)
Step 3: Robust Z-score normalization	All retained numeric features (GPS, accuracy, quaternion, terrain, drift)	Robust scaling using median and IQR: $\left(x-\tilde{x}\right)/\left(\mathrm{IQR}+\varepsilon \right)$	Scaled feature matrix $X_{\mathrm{scaled}}$
Step 4: C-SMOTE balancing	Scaled feature matrix $X_{\mathrm{scaled}}$ and class label *y*	k-means clustering on minority class; intra-cluster interpolation $x_{\mathrm{new}}=\alpha x_{i}+\left(1-\alpha \right)x_{j}$	Class-balanced training set (synthetic minority samples)
Step 5: Kalman GPS denoising	GPS positional axes (applied to one stream used for refinement): {lat_y, lon_y, alt_y}	Kalman filter prediction-update recursion; replace raw axes with filtered estimates	Denoised GPS trajectory features
Step 6: Quaternion smoothing	Orientation quaternion channels: q_0, q_1, q_2, q_3	Savitzky–Golay smoothing (p=2, w=11) to reduce jitter while preserving dynamics	Smoothed quaternion channels ${\hat{q}}_{0}$–${\hat{q}}_{3}$ (or replaced channels)
Step 7: Feature audit/selection	Candidate features after Steps 1–6; domain-critical: timestamp, q_0–q_3, GPS accuracy (eph_x, epv_x), altitude/terrain (alt_x, alt_ellipsoid_x, terrain_alt)	Pearson correlation with label *y*; keep $|r_{i}|\;\geq \;\delta$ plus domain-critical features	Final feature vector $x^{⋆}$ (Table 2)
Learning (DARTS-ANN)	Final feature vector $x^{⋆}$ (multi-sensor telemetry features) + label *y* for training	Differentiable architecture search; lightweight ANN selection and training for onboard inference	Predicted spoofing state $\hat{y}$; confidence score (if used)
Encryption + authentication (PRESENT-128 + CMAC)	C2 message payload (e.g., navigation/actuation commands and control frames) and session key material	Encrypt payload with PRESENT-128 (CBC) and authenticate using CMAC	Confidential + integrity-protected C2 packets (sensor measurements remain unchanged)

**Table 5:** Performance metrics of machine learning classifiers in Experiment 1 (training and testing phases).

Classifier	Accuracy (%)	Precision	Recall	F1-Score
**Training Phase**				
Fine Decision Tree	97.40	0.972	0.974	0.973
Linear SVM	99.55	0.999	0.999	0.999
Coarse KNN	94.68	0.938	0.946	0.942
Quadratic Discriminant Analysis	99.49	0.997	0.997	0.997
Naive Bayes (Gaussian)	96.82	0.958	0.968	0.963
**Testing Phase**				
Fine Decision Tree	96.99	0.965	0.970	0.967
Linear SVM	99.60	0.999	1.000	0.999
Coarse KNN	94.30	0.935	0.943	0.939
Quadratic Discriminant Analysis	99.53	0.996	0.997	0.997
Naive Bayes (Gaussian)	96.30	0.958	0.961	0.960

**Table 6:** Performance results of DARTS-based ANN models across five runs.

Run ID	Phase	Accuracy (%)	Precision	Recall	F1-Score
DARTS-1	Training	98.68	0.986	0.987	0.986
	Testing	98.81	0.987	0.988	0.987
DARTS-2	Training	98.89	0.988	0.989	0.988
	Testing	98.97	0.989	0.990	0.989
DARTS-3	Training	99.21	0.992	0.992	0.992
	Testing	99.35	0.993	0.994	0.993
DARTS-4	Training	99.66	0.996	0.997	0.996
	Testing	99.745	0.997	0.997	0.997
DARTS-5	Training	99.98	0.999	0.999	0.999
	Testing	99.99	1.000	0.999	0.999

**Table 7:** Per-run experimental results with mean ± standard deviation (SD) for the encryption and secure communication layer. Each block lists five experimental runs, followed by the aggregated mean ± SD for that metric.

Algorithm	Metric	Run ID	Value	Unit
PRESENT-128	Encryption Time	Run 1	0.83	ms
		Run 2	0.86	ms
		Run 3	0.81	ms
		Run 4	0.85	ms
		Run 5	0.82	ms
	**Mean ± SD**		**0.834 ± 0.019**	ms
	Decryption Time	Run 1	0.84	ms
		Run 2	0.82	ms
		Run 3	0.83	ms
		Run 4	0.85	ms
		Run 5	0.81	ms
	**Mean ± SD**		**0.83 ± 0.015**	ms
	CMAC Verification Time	Run 1	0.12	ms
		Run 2	0.13	ms
		Run 3	0.11	ms
		Run 4	0.12	ms
		Run 5	0.12	ms
	**Mean ± SD**		**0.12 ± 0.007**	ms
	Total Latency Overhead	Run 1	1.78	ms
		Run 2	1.80	ms
		Run 3	1.81	ms
		Run 4	1.77	ms
		Run 5	1.79	ms
	**Mean ± SD**		**1.79 ± 0.015**	ms
	Throughput	Run 1	382.1	kb/s
		Run 2	384.0	kb/s
		Run 3	381.2	kb/s
		Run 4	383.1	kb/s
		Run 5	381.6	kb/s
	**Mean ± SD**		**382.4 ± 1.0**	kb/s
	Energy Consumption	Run 1	0.50	mJ
		Run 2	0.53	mJ
		Run 3	0.51	mJ
		Run 4	0.49	mJ
		Run 5	0.52	mJ
	**Mean ± SD**		**0.51 ± 0.015**	mJ
AES-128	Encryption Time	Run 1	1.41	ms
		Run 2	1.39	ms
		Run 3	1.43	ms
		Run 4	1.46	ms
		Run 5	1.44	ms
	**Mean ± SD**		**1.43 ± 0.025**	ms
	Decryption Time	Run 1	1.37	ms
		Run 2	1.40	ms
		Run 3	1.35	ms
		Run 4	1.39	ms
		Run 5	1.36	ms
	**Mean ± SD**		**1.37 ± 0.018**	ms
	CMAC Verification Time	Run 1	0.21	ms
		Run 2	0.22	ms
		Run 3	0.20	ms
		Run 4	0.21	ms
		Run 5	0.23	ms
	**Mean ± SD**		**0.21 ± 0.011**	ms
	Total Latency Overhead	Run 1	3.00	ms
		Run 2	3.05	ms
		Run 3	2.98	ms
		Run 4	3.02	ms
		Run 5	3.01	ms
	**Mean ± SD**		**3.01 ± 0.025**	ms
	Throughput	Run 1	262.3	kb/s
		Run 2	261.1	kb/s
		Run 3	260.9	kb/s
		Run 4	263.5	kb/s
		Run 5	261.2	kb/s
	**Mean ± SD**		**261.8 ± 1.0**	kb/s
	Energy Consumption	Run 1	0.86	mJ
		Run 2	0.88	mJ
		Run 3	0.85	mJ
		Run 4	0.90	mJ
		Run 5	0.87	mJ
	**Mean ± SD**		**0.87 ± 0.017**	mJ
ChaCha20	Encryption Time	Run 1	1.07	ms
		Run 2	1.09	ms
		Run 3	1.05	ms
		Run 4	1.10	ms
		Run 5	1.08	ms
	**Mean ± SD**		**1.08 ± 0.018**	ms
	Decryption Time	Run 1	1.03	ms
		Run 2	1.04	ms
		Run 3	1.06	ms
		Run 4	1.05	ms
		Run 5	1.02	ms
	**Mean ± SD**		**1.04 ± 0.015**	ms
	CMAC Verification Time	Run 1	0.19	ms
		Run 2	0.18	ms
		Run 3	0.20	ms
		Run 4	0.19	ms
		Run 5	0.19	ms
	**Mean ± SD**		**0.19 ± 0.007**	ms
	Total Latency Overhead	Run 1	2.65	ms
		Run 2	2.70	ms
		Run 3	2.68	ms
		Run 4	2.67	ms
		Run 5	2.65	ms
	**Mean ± SD**		**2.67 ± 0.018**	ms
	Throughput	Run 1	298.1	kb/s
		Run 2	301.0	kb/s
		Run 3	297.6	kb/s
		Run 4	300.2	kb/s
		Run 5	299.8	kb/s
	**Mean ± SD**		**299.3 ± 1.3**	kb/s
	Energy Consumption	Run 1	0.75	mJ
		Run 2	0.78	mJ
		Run 3	0.76	mJ
		Run 4	0.77	mJ
		Run 5	0.74	mJ
	**Mean ± SD**		**0.76 ± 0.015**	mJ

**Table 8:** Comparison of GPS spoofing detection studies with respect to deployment feasibility and operational characteristics.

Study	Platform/Setup	Latency	Energy	Model Size	Operational Evaluation
[18]	Real UAV flights (Pixhawk 2.4.8, GPS+IMU+Barometer)	1.94	Not reported	Not reported	Real flight logs (single missions)
[18]	PX4 UAV + u-blox M8 + SDR spoofing	1.81 ms	Not reported	Traditional ML	Outdoor flights (static/dynamic)
[26]	Small UAV (TinyML-targeted, offline evaluation)	1.84 ms	Not reported	64–32–4 DNN	Offline; no sustained duration
[23]	Small UAV, controlled simulation	Not reported	Not reported	Stacked ensemble	Simulation-based
[22]	CARLA autonomous vehicle simulator	2.13 ms	Not reported	CNN/SVM	Simulation trajectories
This work	Edge UAV system (multi-sensor telemetry)	1.79 ms	0.51 mJ	DARTS-ANN	Real-time telemetry

## Data Availability

The dataset used in this study is the UAV Attack Dataset, which is publicly available from IEEE DataPort. The dataset can be accessed at https://ieee-dataport.org/open-access/uav-attack-dataset (accessed on 8 January 2026). All experimental analyses were conducted using this dataset. Additional processed data and scripts generated in this work are available from the corresponding author upon reasonable request.
